# Anti-Biofilm Activity of Chlorogenic Acid against *Pseudomonas* Using Quorum Sensing System

**DOI:** 10.3390/foods12193601

**Published:** 2023-09-28

**Authors:** Lin Wang, Xueli Cao, Hairun Pei, Ping Liu, Ya Song, Yulun Wu

**Affiliations:** 1 Beijing Advanced Innovation Centre for Food Nutrition and Human Health, Beijing Technology and Business University, Beijing 100048, China; linwang1110@163.com (L.W.); 13619506127@163.com (P.L.); 17861406937@163.com (Y.S.); 13333433543@163.com (Y.W.); 2 School of Light Industry, Beijing Technology and Business University, Beijing 100037, China

**Keywords:** chlorogenic acid, *Pseudomonas putida*, biofilm, quorum sensing, Las, Pqs, Rhl

## Abstract

Chlorogenic acid is a secondary metabolite produced by many traditional Chinese medicines. Its physiological activities (antibacterial, anti-inflammatory, antioxidant activities, etc.) have been well described. This study aimed to investigate the effects of chlorogenic acid on the biofilm of drinking water bacteria. The effects of chlorogenic acid on the metabolites of the biofilms were also evaluated. Chlorogenic acid was found to have an anti-biofilm effect against *Pseudomonas*, resulting in biofilm formation in a dose-dependent manner (0.53–25.4 mM CGA). Moreover, the biofilm structure was visibly attenuated. Furthermore, we identified and characterized 23 differential metabolites and associated two metabolic pathways involving beta-alanine metabolism and pyrimidine metabolism that were altered mostly during biofilm formation. A quantitative real-time PCR assay revealed that chlorogenic acid interfered with the signaling molecule synthesis and transcription regulators using the Las, Pqs and Rhl systems. These findings suggest that chlorogenic acid can be a quorum sensing (QS) inhibitor and inhibit biofilm formation. It may be a promising natural product for the prevention of contaminated drinking water.

## 1. Introduction

Plastic-packaged drinking water (PPDW) refers to water that is sealed in polycarbonate packaging containers that comply with food safety standards and relevant regulations and is available for direct consumption, including purified drinking water and mineral water [1]. PPDW emerged in China in the mid-1990s and has gained popularity for making tea, drinking and cooking [2]. Therefore, consumers are increasingly paying attention to its safety requirements. However, microbial contamination in reusable PPDW is relatively serious [3,4,5]. *Pseudomonas* is the main opportunistic bacterial pathogen in PPDW [6]. 

Cells tend to adhere to surfaces and form gelatinous films, termed biofilms, during the growth process. These biofilms often contribute to corrosion and fouling processes in water transportation, storage and treatment [7,8]. Biofilms provide an easily attachable surface for many microorganisms, which often leads to a decrease in water quality. Once the biofilm is established in a water pipe network or on pipeline surfaces, it is difficult to remove and leads to a decline in water quality [9,10,11]. Particularly, in summer, with an increase in water temperature and a reduction in disinfectant levels in pipelines, microorganisms can transfer into water from biofilms and rapidly reproduce [7]. 

Biofilms are regulated by QS systems [12]. QS is a system of stimuli and responses in relation to bacterial cell population density that regulates gene expression, including virulence determinants [13]. Las, Rhl and Pqs are the main systems that regulate the virulence genes of *Pseudomonas* [14]. All three systems comprise a diffusible autoinducer signaling molecule and a regulator protein [15]. When population density is high, the autoinducer binds to its cognate receptor to form a complex that regulates gene transcription [14].

*P. aeruginosa* is recognized as the main pathogen that frequently appears in PPDW [9,16]. The EU Drinking Water Quality Directive (98/83/EC) also states that the index value of *P. aeruginosa* in bottled or barked drinking water is 0 PCS/250 mL. The United States, Canada, Brazil and Japan restrict the most probable number of *P. aeruginosa* in bottled drinking water (including natural mineral water sources) to <3/L or undetectable in every 250 mL [17]. It has been used as a model bacterium to study its pathogenic mechanism in drinking water systems [18]. However, there are few studies on other species of *Pseudomonas* as pathogenic bacteria in drinking water systems. The presence and composition of *Pseudomonas* and biofilm-forming *Pseudomonas* strains in PPDW are still insufficiently known. Furthermore, there is little correlation between QS genes and bacterial metabolites.

Chlorogenic acid (CGA) is a polyphenol compound [19]. It is a secondary phenolic metabolite produced by tea, coffee, etc. [20]. With its structure of a benzene ring, multiple hydroxyl groups and polyunsaturated hydrocarbons, CGA participates in the diversification of biochemical reaction pathways and has various antimicrobial properties that make it a potential candidate for use as a preservative and food additive [19,21]. Recent works have suggested that CGA can inhibit the biofilm formation, aggregation and quorum sensing (QS) systems of *Pseudomonas* [22,23]. On the other hand, CGA is practically non-degradable in the presence of probiotics, which makes it appropriate for use in the food industry [24]. 

This study aimed to investigate the anti-biofilm effect of CGA against *Pseudomonas* biofilm. Our focus was on identifying the distinct metabolites affected by CGA in *Pseudomonas* biofilm and the metabolic pathways influenced by the QS system. 

## 2. Materials and Methods

### 2.1. Growth Condition of Strains

Bacterial strains were isolated from commercial plastic-packaged drinking water. The identification of the strains is listed in Table S1. All strains were kept in our laboratory and propagated in Luria–Bertani medium (LB) (Luqiao Co., Ltd., Beijing, China) at 36 °C. CGA was purchased from Zelang Biosciences Inc. (Nanjing, China), with a purity of ≥98%. The isolation and identification of strains in samples were carried out according to the “57 *P. aeruginosa*” method in GB 8538-2016 “*National Standard for Inspection of Natural Mineral Water for Drinking*”, and the method was slightly modified. Colonies with typical biochemical characteristics were validated by BioMerieux’s automatic microbial identification system (VITEK 2 COMPACT, Lyons, France).

### 2.2. Minimum Inhibitory Concentration Assays

The minimum inhibitory concentration (MIC) of CGA against *Pseudomonas* was determined using microdilution methods, following a procedure with some modifications, as described below [25]. The CGA stock solution with a concentration of 200 mM stored in LB was serially added to the sterile LB broth in a 96-well round-bottom plate. Then, 95.00, 85.00, 75.00, 65.00, 60.00, 50.00, 40.00, 33.00, 25.00, 15.00, 8.00, 4.00, 2.00, 1.00, 0.50 and 0.00 mM CGAs in an LB culture medium were obtained. Each well contained 190 μL of serially diluted CGA, to which 10 μL of the diluted culture was added before incubation at 36 °C for 24 h. Five parallel samples were prepared at each concentration, and LB without CGA was used as the control. 

### 2.3. Biofilm Formation Ability

The biofilm formation ability of 23 isolated strains was determined using the crystal violet (CV) method [26]. After 12 h of incubation at 36 °C, bacterial suspension was adjusted with LB to an initial optical density of 10^7^ CFU/mL. Then, 10 μL was added to a 96-well polystyrene microtiter plate with 190 μL LB in each well, and the well containing a blank medium without bacteria was used as the control. After 24 h of incubation at 36 °C, the cells were removed. The biofilm was washed three times with 200 μL phosphate-buffered saline (PBS, pH 7.4) to remove planktonic and loosely bound cells. The adherent cells were fixed with 200 μL anhydrous methanol. After fixing, 200 μL 0.1% crystal violet was added to stain each well for 30 min. After rinsing every well with tap water for 3 s, 200 μL of 33% acetic acid was used to dissolve the stained biofilm. The absorbance was measured at 595 nm.

### 2.4. Biofilm Inhibition and Removal Assay

The overnight bacterial culture of *Pseudomonas* with the best capability for biofilm formation was diluted to 10^7^ CFU/mL in fresh LB medium. For inhibition groups, 10 µL of the diluted culture was mixed with 190 µL of varying concentrations of CGA (sub-MIC, 1/2 sub-MIC, 1/4 sub-MIC, 1/8 sub-MIC, 1/16 sub-MIC and 1/32 sub-MIC) in 96-well polystyrene microtiter plates and incubated at 36 °C for 24 h. A well containing a blank medium without CGA was used as the non-treated control. For biofilm removal, the LB culture was gently aspirated after biofilm formation, and 200 µL solutions with different concentrations of CGA in LB replaced the culture medium and were incubated at 36 °C for 24 h. The absorbance was measured at 595 nm.

### 2.5. Microscopic Investigation

Biofilm was also observed using scanning electron microscopy (SEM). Discs (each square disc had 1.8 cm × 1.8 cm dimensions) were placed in a 24-well plate and incubated with bacteria, as mentioned above. After 16 h, the discs were removed from the tubes and placed in a sterile PBS (phosphate-buffered saline, pH 7.4) solution. Then, the discs were gently rinsed with PBS and fixed with 2.5% glutaraldehyde (2.5%, *v*/*v*, Yuanye Co., Ltd., Nanjing, China) at 4 °C for 24 h. After glutaraldehyde fixation, the discs were washed again with PBS for 1 h and dehydrated by increasing the concentrations of ethanol. Specimens were laid on stubs and coated with gold–palladium. Digital images were obtained using SEM (SU8020, 3KV/5KV accelerating voltage; Hitachi, Japan) examination. To compare the biofilm inhibition, discs without CGA were used as the control.

### 2.6. FTIR Analysis

The obtained biofilms of CGA-treated (25.40, 16.93, 8.47 mM) and untreated samples were washed and immobilized with glutaraldehyde (2.5%, *v*/*v*) at 4 °C for 24 h. The fixed cells were then serially dehydrated using ethanol (from 50% to 90%). Then, 1 mg bacterial cells and 100 mg KBr were mixed, ground and pressed [27]. The FTIR spectra were tested using an FTIR spectrophotometer (NICOLET iS10, Thermo Fisher, Waltham, MA, USA).

### 2.7. Screening of CGA

The suspensions in the culture medium (2.4) were centrifuged at 4571× *g* (2000 rpm in an F21-48 × 1.5/2.0, Model RPM centrifuge, Thermo Scientific Co., Shanghai, China) at 4 °C for 15 min to remove the planktonic cells. Cell-free supernatant was obtained through a 0.22 μm filtration membrane.

To assay the content of CGA, UPLC separation was performed on a Waters Acquity UPLC-I-Class system (Waters Corporation, Milford, MA) using a Waters ACQUITY UPLC HSS T3 1.8 µm (2.1 mm×100 mm) analytical column; mobile phase A was water with 0.1% formic acid (*v*/*v*), and mobile phase B was acetonitrile with 0.1% formic acid (*v*/*v*) with an optimized gradient-elution program: 0–2 min, 0–95% B; 2–9 min, 95–50% B; 9–15 min, 50–10% B; 15–17 min, 90–90% B; 17–19 min, 90–5% B; 19–22 min, 5–5% B [28]. The flow rate for separation systems was set at 0.4 mL/min, and the column temperature was maintained at 40 °C. All the samples were placed at 4 °C with a 1 μL injection. The parameters of MS acquisition are summarized as follows: desolvation temperature, 25 °C; cone gas flow, 50 L/h; desolvation gas flow, 600 L/h; capillary voltage, 2500 V in negative mode; source temperature, 100 °C; MassLynx software was used for fragment analysis to determine the structure of the compound.

### 2.8. Metabolite Extraction and GC-MS Analysis

After 24 h, the culture flasks were kept on ice, and the biofilms were isolated from 24-well cell culture plates (REF 3524, Corning, NY, USA). The suspensions in the culture medium were removed. The biofilms were further washed with 2 mL of PBS 3 times, and such a procedure was always maintained on ice. Then, 2 mL of 80% ice-cold methanol was added, and the biofilm was scraped off with a cell scraper. The solution was homogenized on ice for 2 min. This procedure was repeated three times. Next, the obtained samples were centrifuged at 20,959× *g* (12,000 rpm in an F15-8 × 50cy rotor, Thermo Scientific Co., Ltd., Shanghai, China) at 4 °C for 15 min. Finally, the supernatants were passed through a 0.22 mm filter membrane (nylon) before nitrogen blowing. The condensed samples were stored at −80 °C for GC-MS-based metabolomics analysis [29].

Samples were analyzed using a GC-MS system (GCMS 7890A-5975C, Agilent Technologies, Santa Clara, CA, USA). Metabolome assay was carried out on a system using helium (≥99.999%) as the carrier gas under constant pressure (injector port pressure: 7.0699 psi). A total of 1 μL was injected into a DB-Wax 30 m × 0.25 mm × 0.25 μm column using splitless injection. The oven temperature was increased from 40 °C (held for 2 min) to 210 °C at 5 °C/min and then increased at 10 °C/min to 240 °C (held for 2 min), for a total run time of 41 min. The injector port, ion source, quadrupole and interface were maintained at 250 °C, 230 °C, 150 °C and 250 °C, respectively. The flow rate was set at 1.0 mL/min. Scan time segments were full scans without solvent delay, with scanning from 40 to 350 *m*/*z*.

### 2.9. RNA Isolation and RT-qPCR 

After 24 h of culture in 16.93 mM CGA in LB, RNAs were extracted and purified [30]. The purified RNAs were reverse-transcribed to cDNAs. Real-time reverse transcription–polymerase chain reaction (RT-qPCR) was performed using an Applied Biosystems ABI7300 (Thermo Fisher, Shanghai, China), in which a ChamQ SYBR Color qPCR Master Mix (Nanjing, China) was used. Primers for target genes were designed for quorum sensing. The 16S rRNA gene was used as the reference for comparison with the target genes. For the sequences, refer to Wenya et al. Primers were synthesized by Ruibo Biotechnology Co., Ltd. (Beijing, China). PCR reactions were performed using the method described by Wenya Xu et al. [31]. The cycling conditions were as follows: 1 cycle at 95 °C for 5 min; 40 cycles for denaturation at 95 °C for 30 s, with annealing at 52–58 °C (depending on the primers used) for 30 s; and extension and fluorescent data collection at 72 °C for 30 s. Data were calculated using the 2-ΔΔCT method by Christian et al. [32].

### 2.10. Statistical Analysis

The results were expressed as mean ± SD. One-way analysis of variance (ANOVA) was performed to compare differences between groups followed by Tukey’s test. A *p*-value below 0.05 was considered statistically significant.

## 3. Results

### 3.1. Pseudomonas Strains Biofilm Formation Ability

**Figure 1:**
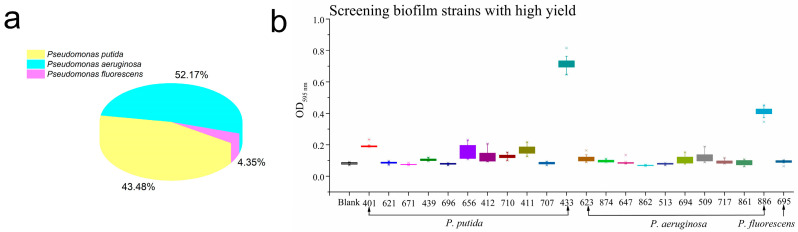
Biofilm formation capabilities of *Pseudomonas*. (**a**) *Pseudomonas* contamination in PPDW. (**b**) Screening biofilm formation ability of strains by CV stain.

All the identified strains are summarized in Table S1, together with their assigned confidence level. Of all the bacteria isolated from PPDW, *P. putida* and *P. aeruginosa* comprised the highest proportion, accounting for 43.48% and 52.17%, respectively (Figure 1a).

The biofilm formation ability of 23 isolated strains was measured. Different strains have different biofilm formation abilities, with the median value of the measured biofilm value of *P. putida* 433 being approximately 0.7 and the outlier value not more than 0.8, which is significantly higher than the other strains (*p* < 0.05). *P. putida* 433 had the strongest biofilm formation ability (Figure 1b). Therefore, *P. putida* 433 was selected for biofilm formation at 36 °C for 24 h for the following experiments.

### 3.2. Antibacterial Activity of CGA against P. putida

**Figure 2:**
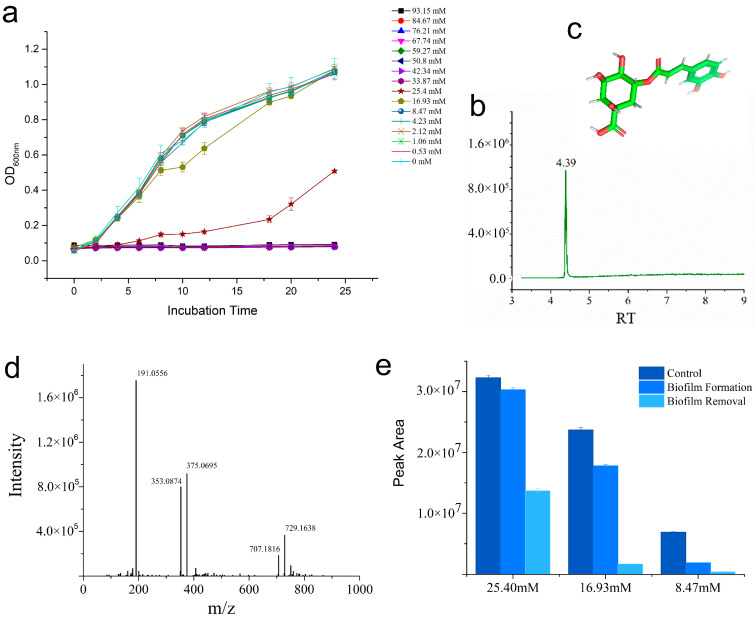
Effects of CGA on the growth of *P. putida* 433. (**a**) Growth curve of *P. putida* 433. (**b**) Chemical structure of CGA. (**c**) Mass identification and chemical structure of CGA. (**d**) Retention time of CGA. (**e**) LC-MS analysis of concentration of CGA in biofilm culture. Dark-blue bars: control groups with 25.4, 16.93 and 8.47 mM CGA, without *P. putida* 433, at 37 °C, 24 h. Mid-blue bars: biofilm formation with 25.4, 16.93 and 8.47 mM CGA, with *P. putida* 433, at 37 °C, 24 h. Light-blue bars: biofilm removal with 25.4, 16.93 and 8.47 mM CGA added to the formed biofilm (37 °C, 24 h), at 37 °C, 24 h. Data are shown as the mean ± SD of triplicate experiments. There are significant differences in different groups at different concentrations, *p* < 0.05.

Figure 2a reveals that CGA has an antibacterial effect on *P. putida* 433 strain, with an MIC value of 25.4 mM, and CGA had no effect on the growth at 16.93 mM (*p* > 0.05). The content of CGA was tested using LC-MS. The chemical structure, mass spectrometry and TIC of CGA are shown in Figure 2b–d, respectively.

### 3.3. Effects of CGA on the Biofilm Formation of P. putida

**Figure 3:**
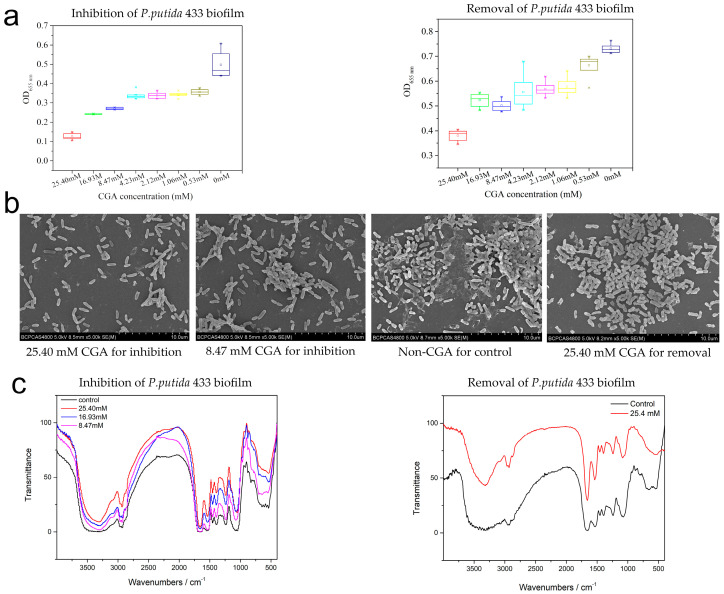
Effect of CGA on *P. putida* 433 biofilm. (**a**) CV staining of biofilm formation and biofilm removal. Data are shown as mean ± SD. There are significant differences for different groups, *p* < 0.05. (**b**) Scanning electron microscope images of biofilm. (**c**) FTIR of biofilm formation and removal.

CGA demonstrated a significant inhibitory effect on the biofilm formation of *P. putida* 433 when used at concentrations above 0.53 mM (1/48 MIC). Biofilm was also removed by CGA (0.53–25.4 mM). Notably, a decrease in the inhibitory and removal effect of CGA on *P. putida* 433 biofilm was correlated with a decrease in CGA concentration (Figure 3a). 

Compared to the control group (without *P. putida*, dark-blue bars), the peak area of CGA in the culture decreased during biofilm formation (mid-blue bars) and removal (light-blue bars) (Figure 2e). It can be implied that CGA was consumed by the bacteria and/or metabolized into active ingredients after entering the biofilm. The peak area of CGA during biofilm removal decreased more than that during biofilm formation (Figure 2e). This indicates that CGA may be involved in biofilm metabolism.

### 3.4. The Effect of CGA on Biofilm Microstructure

Microstructure analysis was carried out using scanning electron microscopy (SEM) under 10 μm scales to visually verify the inhibitory effects of CGA on biofilms. In the control group (non-CGA), the biofilm covered the entire field of vision, and the bacteria were densely arranged with complete morphology. In the culture of 25.40 mM (1 MIC) CGA in LB, it had a weak capacity for biofilm formation, a relatively dispersed distribution and a low degree of bacterial aggregation (Figure 3b). In the culture of 8.47 mM (1/2 sub-MIC) in LB, the degree of bacterial aggregation in the structure was higher. Compared to the control group, a loose biofilm and less EPS microstructure were exhibited in the removal group. CGA suppressed biofilm maturation and affected the structure of the mature biofilm. 

FTIR spectra of the non-treated control and CGA-treated biofilm were measured to determine changes in biofilm components, as shown in Figure 3c. The black line is non-CGA-treated *P. putida* 433 biofilm spectrum and reveals characteristic peaks of polysaccharides, such as a broad peak at 3600–3100 cm^−1^ assigned to the hydration of exopolysaccharides (EPSs) and bacterial cells, a peak at 3000–2800 cm^−1^ corresponding to the C–H of EPS and a peak at 1800–1600 cm^−1^ corresponding to amide linkages within proteins and peptides, indicating the protein matrix in the biofilm. The peak at 1600–1500 cm^−1^ can be assigned to the C=O of polysaccharides. Peaks of 1500–1000 cm^−1^ were mainly caused by a variable angle vibration of C–H. Peaks at 1200–1000 cm^−1^ were caused by two kinds of C–O stretching vibrations. As CGA increased, peaks of CGA inhibition groups relatively narrowed at 3600–3100 cm^−1^, 1800–1600 cm^−1^ and 1600–1500 cm^−1^. This indicates that the metabolism of biofilm changed according to CGA. 

### 3.5. Changes in Metabolites of P. putida Biofilms Caused by CGA

**Figure 4:**
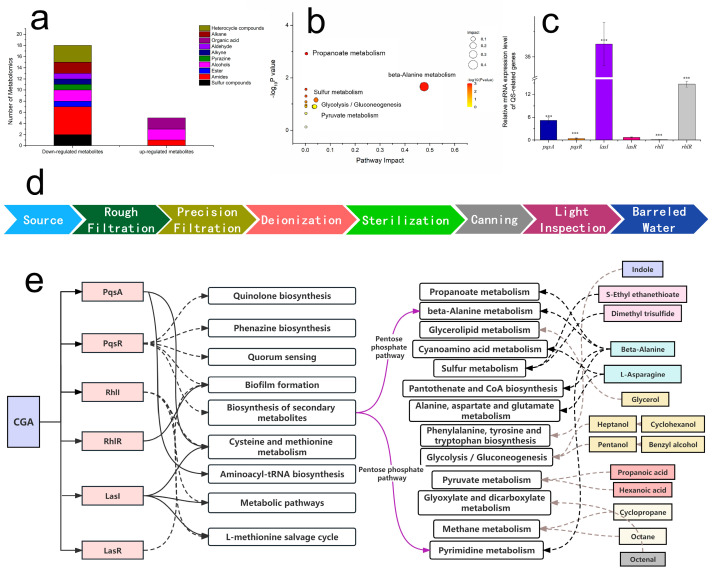
Changes in metabolites and QS-related genes in *P. putida*. (**a**) Metabolomics of down-regulated and up-regulated compounds. Compared to the non-CGA-treated groups, the contents of 19 compounds (dimethyl trisulfide, ethyl ethanethioate, tartaramide, methanethioamide, propanamine, methyl aminoacetate) were relatively low. (**b**) Bubble map of metabolic pathways. (**c**) Effects of CGA on the gene expression of QS regulatory circuits. *** means that there are significant differences in different genes, *p* < 0.05. (**d**) Processing flow chart of PPDW. (**e**) The analysis of possible mechanisms affected by CGA. Up-regulations are represented by a solid line, and down-regulations are represented by a dotted line. The dashed gray lines indicate that there is a correlation between metabolites and pathways, but they do not play an up-regulation or down-regulation role in this study.

To investigate the change in the metabolites of biofilms caused by CGA, GC-MS analysis was carried out to analyze the secretions of biofilm intercellular metabolites. The types of metabolites were identified based on the NIST 14 Mass Spectral Library, and the metabolite names, formulas and peak areas are listed in Table S2. The types of differential compounds from *P. putida* 433 were classified as sulfur, amide, ester, alcohol, pyrazine, alkyne, aldehyde, organic acid, alkane and heterocycle compounds (Figure 4a). Of them all, the peak area of amide, alcohol and heterocycle compounds changed most obviously. Heterocycle, alkane, aldehyde, alkyne, pyrazine, ester and sulfur compounds were down-regulated, while organic acids were up-regulated. However, most amides were down-regulated, with only isobutylamine up-regulated. Compared to the non-CGA-treated groups, the contents of 19 compounds (dimethyl trisulfide, ethyl ethanethioate, tartar-amide, methane-thioamide, propanamide, methyl amino acetate, aminoacethydrazide, octanethioic acid methyl ester, pentanol, benzyl alcohol, methyl pyrazine, heptyne, octenal, cyclopropane, 2,2-dimethyl-aziridine, 1,6-octadiene and thiapyran) were up-regulated (Table S2). CGA affected multiple components of the biofilm.

### 3.6. CGA-Regulated QS-Related Genes

The expression of QS-related gene regulation in *P. putida* 433 biofilms was examined to clarify the regulatory effect of CGA on the QS system at the mRNA expression level. The expression of these genes was evaluated, and their Ct values were calculated to determine the relative expression. The amplified and melting curves of relevant genes in both CGA-treated and untreated strains are shown in Figure 4c. The expression of pqsR, rhlL and lasR genes were down-regulated. On the contrary, the CGA-treated group showed a remarkably changed gene expression level of *pqsA*, *lasI* and *rhlR* (*p* < 0.05). QS is crucial for biofilm formation [33]. The Las, RhL and Pqs systems can trigger the production of biofilm virulence factors (pyocyanin and elastase) and were the most highlighted QS systems in previous studies. The Las and RhL systems use acyl homoserine lactones (AHLs) as a signaling molecule, which binds to the receptor proteins of LasR and RhlR and regulates extracellular enzymes and rhamnolipids, respectively [34,35]. The Pqs system uses another kind of molecule to promote the release of eDNA [36,37]. The expression level of the synthesis of QS signals—*lasI*, *rhlI* and *pqsA*—and transcription regulators—*lasR*, *rhlR* and *pqsR*—was investigated. Then, the effects of CGA on the above three QS systems were confirmed. The transcription of signaling molecule synthesis genes *lasI* in the Las system and pqsA in the Pqs system was up-regulated, while *rhlI* in the RhL system was down-regulated. The transcription of receptor genes *lasR* in the Las system and *pqsR* in the Pqs system was down-regulated, while *rhlR* in the RhL system was up-regulated. In summary, CGA influences biofilm formation by interfering with transcription regulators and signaling molecule synthesis (Figure 4e). Several studies have found that the suppressed effects were all involved in the transcriptional inhibition of genes controlling the syntheses and secretions of exopolysaccharides, extracellular proteins and virulence factors [38,39]. This study further confirms that CGA affects the Las, Rhl and Pqs systems.

## 4. Discussion

### 4.1. Risk Analysis of Pseudomonas Contamination

PPDW is usually obtained through various treatment methods, such as tap water or groundwater extraction, reverse osmosis [40], electrodialysis, distillation [41], softening and other treatment modes [5]. The general technological flow of packaged drinking water (Figure 4d) involves several critical steps [42,43]. The filtration system and reverse osmosis system are the main links causing *Pseudomonas* pollution [44]. When raw water passes through coarse filtration and fine filtration equipment, as well as a two-stage reverse osmosis system, the detection rate of *Pseudomonas* increases significantly [45], indicating that certain equipment in the production line may not be functioning optimally, or the filtration membrane and other devices are not replaced in time, which increases the *Pseudomonas* contamination rate. Because the geographical location of water sources or distances from living areas are different, the degree of microbial pollution is different [4,6,46,47]. According to FDA standards, *P. aeruginosa* cannot be detected. However, there are no restrictions on other species of *Pseudomonas*. Concerns regarding the presence of *Pseudomonas* in PPDW are growing as the combination of suitable temperatures [6], low flow rates and poor nutrients makes PPDW an ideal environment for *Pseudomonas* proliferation and biofilm formation [48]. 

### 4.2. Anti-Biofilm Effect of CGA on P. putida

A biofilm is a collective community of microorganisms. It possesses many emergent characteristics, including interface aggregation, surface adhesion, water retention, nutrient absorption and retention, enhanced resistance and cooperation [49]. Once formed, the bacterial biofilm becomes a shelter for bacteria, which significantly enhances the resistance of bacteria to antimicrobial agents [50]. Therefore, the inhibition of biofilm formation, especially those of *P. putida*, is important for the safety of PPDW.

Given the negative effects of antimicrobial agents, phytochemicals have been recognized as a promising substitute [17,48]. CGA can inhibit the biofilm formation of *P. putida* by inhibiting bacterial growth. This phenomenon is attributed to CGA passing the extracellular matrix or being involved in biofilm metabolism. CGA is rapidly metabolized into active ingredients, and the bioavailability of CGA largely depends on its metabolites [51]. According to recently published research, quinic acid and dihydrocafeic acid are the main metabolites of CGA, which could eliminate the biofilm produced by *P. aeruginosa* at sub-MICs ranging from 1/3 MIC to 1 MIC and 1/128 MIC to 1/2 MIC; quinic acid could exhibit potentially greater inhibitory effects on biofilm formation [52]. Additionally, many plant-derived anti-biofilm agents are chemical entities endowed with uniquely diverse features, including complex structures that could not be easily obtained via a synthetic pathway, potentially providing a completely different array of action mechanisms when compared with antibacterials [53].

In our previous study, honeysuckles could destroy the structure of mature biofilms, but the existence of intermolecular forces within the biofilm matrix (EPS, protein, eDNA, water, etc.) and structure remains unknown [54]. LC-MS data showed that CGA in culture decreased compared with the control group without the biofilm (Figure 2e), which is consistent with previous findings that showed CGA was metabolized into active ingredients after entering the biofilm. Other studies also found that CGA at 256 μg/mL (0.72 μM) provided a maximum 80% inhibition of *P. aeruginosa* biofilm formation (80–256 μg/mL) [55]. The discrepant values observed can be attributed to variations in the biofilm formation ability of bacteria and the different culture systems used in the experiment. In this study, we innovatively assayed the content of CGA in the culture of biofilm to prove the function of biofilm inhibition. 

### 4.3. Beta-Alanine and Pyrimidine Metabolism Were Affected by CGA through QS System

CGA has been found to impede biofilm formation by restraining many pathways [56,57]. However, the specific regulated pathway remains unclear. Through an enrichment analysis of the pathways, where the differential metabolites were located, the pathways were further screened, as shown in Figure 4b. The important pathways with the highest correlation with metabolite differences were identified. Beta-alanine metabolism showed significant enrichment (*p* < 0.05), and five metabolites were mapped to beta-alanine metabolism. 

As PqsR is down-regulated by CGA, the biosynthesis of secondary metabolites is affected. Then, pyrimidine metabolism is affected through the pentose phosphate pathway [58]. From KEGG, we observe that uracil is the by-product of pyrimidine metabolism and changes to 5,6-dihydrouacil and N-carbamoyl-beta-alanine. Then, beta-alanine is generated by beta-ureidopropionase [59]. Therefore, beta-alanine production decreases. Beta-alanine is not only the key metabolite in beta-alanine metabolism, but also affects propanoate and pyrimidine metabolism, while beta-alanine metabolism and pyrimidine metabolism are affected by the biosynthesis of secondary metabolites through the pentose phosphate pathway. However, pathways of quinolone biosynthesis, phenazine biosynthesis, quorum sensing and secondary metabolism biosynthesis are down-regulated by PqsR.

Other metabolites were analyzed in the same way. Uracil is assigned to pyrimidine degradation [60]. Dihydropyrimidine dehydrogenase is the first member of the NADH-dependent subclass of iron–sulfur flavoenzymes, catalyzing the conversion of uracil to 5,6-dihydrouracil [61]. Beta-alanine and N-Carbamoyl-beta-alanine are produced by two cyclic amidohydrolase enzymes: allantoinase and phenylhydantoinase [62]. In this pathway, the gene products are identified as dihydropyrimidine dehydrogenase (PydA), dihydropyrimidinase (PydB) and beta-alanine synthase (PydC) [63,64].

Overall, CGA may have down-regulated the recognition protein RhlL, but the LasI and PqsA over-expressed to compensate for the reduction in RhlL. As CGA or its metabolites enter the cell, the transcription regulator PqsR is down-regulated. Then, the production enzyme of secondary metabolites biosynthesis decreases. Beta-alanine and pyrimidine metabolism are corresponding effects. However, there are still many unsolved pathways that need to be studied further.

The mechanism governing biofilm formation is a complex process that involves many factors (QS, protease, signal transduction, etc.). The regulation of genes is the most understood mechanism in the inhibition of biofilm formation. There are many other mechanisms involved in biofilm inhibition, and the present regulation of QS by CGA supports the specific QS genes that have been widely reported. On the other hand, natural products from plants have sophisticated defense mechanisms and are rich sources of anti-biofilm agents.

## 5. Conclusions

This study discovered that *P. putida* was also the primary pathogen of PPDW. CGA inhibited the biofilm formation of *P. putida* via changing the structure and weak interaction forces of biofilms as well as interfering with the Las, RhL and Pqs systems. As a result, the QS systems affected multiple metabolites, including beta-alanine and pyrimidine metabolism.

Consequently, CGA exhibits potential as a novel phytochemical candidate for the prevention of *Pseudomonas* contamination. This study also offers new evidence regarding the possible effectiveness of CGA in equipment and environmental applications in the food industry. 

## Data Availability

The data that support the findings of this study are available from the corresponding author upon reasonable request.

## Supplementary Materials

The following supporting information can be downloaded at: https://www.mdpi.com/article/10.3390/foods12193601/s1, Table S1: Results of VITEK Biochemical Identification; Table S2: Peak areas of metabolites by GC-MS analysis.

## References

[1] Bao X., Luo Z., Zhou Z. (2019). Laws and Regulations of Packaged Drinking Water from Both Domestic and Abroad. Beverage Ind..

[2] Liao Z., Cao D., Zhang H., Zhao X. (2017). Current situation and existing problems of packaged drinking water industry in China. J. Food Saf. Qual..

[3] Pu J., Fukushi K. (2016). Bacterial water quality and risk evaluation of bottled drinking water in China. Int. J. Food Sci. Nutr..

[4] Hubbard A.T.M., Newire E., Botelho J., Reiné J., Wright E., Murphy E.A., Hutton W., Roberts A.P. (2020). Isolation of an antimicrobial-resistant, biofilm-forming, *Klebsiella grimontii* isolate from a reusable water bottle. Microbiologyopen.

[5] Mills K., Golden J., Bilinski A., Beckman A.L., McDaniel K., Harding A.S., France A., Tobar H.N., Vecitis C. (2018). Bacterial contamination of reusable bottled drinking water in Ecuador. J. Water Sanit. Hyg. Dev..

[6] Zhou Q., Huang J., Guo K., Lou Y., Wang H., Zhou R., Tang J., Hou P. (2023). Spatiotemporal distribution of opportunistic pathogens and microbial community in centralized rural drinking water: One year survey in China. Environ. Res..

[7] Aloraini S., Alum A., Abbaszadegan M. (2023). Impact of Pipe Material and Temperature on Drinking Water Microbiome and Prevalence of *Legionella*, *Mycobacterium*, and *Pseudomonas* Species. Microorganisms.

[8] Miranzadeh M.B., Mhsanifar E., Iranshahi L. (2011). Evaluation of Bacterial Quality and Trace Elements Concentrations in 25 Brands of Iranian Bottled Drinking Water. J. Agric. Environ. Sci..

[9] Wang X., Wang J., Liu S., Guo J., Fang F., Chen Y., Yan P. (2023). Mechanisms of survival mediated by the stringent response in *Pseudomonas aeruginosa* under environmental stress in drinking water systems: Nitrogen deficiency and bacterial competition. J. Hazard. Mater..

[10] Maes S., Vackier T., Huu S.N., Heyndrickx M., Steenackers H., Sampers I., Raes K., Verplaetse A., De Reu K. (2019). Occurrence and characterisation of biofilms in drinking water systems of broiler houses. BMC Microbiol..

[11] Borjac J., Zeino W., Matar A., Khawaja S., Merheb M., Matar R. (2023). Prevalence of Antibiotic-Resistant Bacteria in Domestic Water Storage Tanks in Sidon, Lebanon. Water..

[12] Tripathi S., Purchase D., Govarthanan M., Chandra R., Yadav S. (2023). Regulatory and innovative mechanisms of bacterial quorum sensing-mediated pathogenicity: A review. Environ. Monit. Assess.

[13] Kalia V.C. (2013). Quorum sensing inhibitors: An overview. Biotechnol. Adv..

[14] Wilder C.N., Diggle S.P., Schuster M. (2011). Cooperation and cheating in *Pseudomonas aeruginosa*: The roles of the *las, rhl and pqs* quorum-sensing systems. ISME J..

[15] McGrath S., Wade D.S., Pesci E.C. (2004). Dueling quorum sensing systems in *Pseudomonas aeruginosa* control the production of the *Pseudomonas* quinolone signal (PQS). FEMS Microbiol. Lett..

[16] Su M., Liu F., Luo Z., Wu H., Zhang X., Wang D., Zhu Y., Sun Z., Xu W., Miao Y. (2019). The Antibacterial Activity and Mechanism of Chlorogenic Acid Against Foodborne Pathogen *Pseudomonas aeruginosa*. Foodborne Pathog. Dis..

[17] Kunpeng G., Juan H.E., Haiyun Z., Han G., Tao Y., Sheng S., Di L. (2022). Analysis of Contamination of *Pseudomonas aeruginosa* in Packaged Drinking Water. China Food Saf. Mag..

[18] Tisler S., Christensen J.H. (2022). Non-target screening for the identification of migrating compounds from reusable plastic bottles into drinking water. J. Hazard. Mater..

[19] Naveed M., Hejazi V., Abbas M., Kamboh A.A., Khan G.J., Shumzaid M., Ahmad F., Babazadeh D., FangFang X., Modarresi-Ghazani F. (2018). Chlorogenic acid (CGA): A pharmacological review and call for further research. Biomed. Pharmacother..

[20] Naso L.G., Valcarcel M., Roura-Ferrer M., Kortazar D., Salado C., Lezama L., Rojo T., González-Baró A.C., Williams P.A.M., Ferrer E.G. (2014). Promising antioxidant and anticancer (human breast cancer) oxidovanadium(IV) complex of chlorogenic acid. Synthesis, characterization and spectroscopic examination on the transport mechanism with bovine serum albumin. J. Inorg. Biochem..

[21] Santana-Gálvez J., Cisneros-Zevallos L., Jacobo-Velázquez D. (2017). Chlorogenic Acid: Recent Advances on Its Dual Role as a Food Additive and a Nutraceutical against Metabolic Syndrome. Molecules.

[22] Rajasekharan S.K., Ramesh S., Satish A.S., Lee J. (2017). Antibiofilm and Anti-Lactamase Activities of Burdock Root Extract and Chlorogenic Acid against *Klebsiella pneumoniae*. J. Microbiol. Biotechnol..

[23] Wang H., Chu W., Ye C., Gaeta B., Tao H., Wang M., Qiu Z. (2019). Chlorogenic acid attenuates virulence factors and pathogenicity of *Pseudomonas aeruginosa* by regulating quorum sensing. Appl. Microbiol. Biotechnol..

[24] Shazly A.B., Fouad M.T., Elaaser M., Sayed R.S., Abd El Aziz M. (2022). Probiotic coffee ice cream as an innovative functional dairy food. J. Food Process Preserv..

[25] Pfaller M.A., Diekema D.J. (2012). Progress in Antifungal Susceptibility Testing of *Candida* spp. by Use of Clinical and Laboratory Standards Institute Broth Microdilution Methods, 2010 to 2012. J. Clin. Microbiol..

[26] Liu N., Li X., Wang M., Zhang F., Wang C., Zhang K., Wang H., Xu S., Hu W., Gu L. (2021). DexA70, the Truncated Form of a Self-Produced Dextranase, Effectively Disrupts *Streptococcus mutans* Biofilm. Front. Microbiol..

[27] Chang A., He Q., Li L., Yu X., Sun S., Zhu H. (2021). Exploring the quorum sensing inhibition of isolated chrysin from *Penicillium chrysogenum* DXY-1. Bioorg. Chem..

[28] Guo R., Lu H. (2020). Targeted metabolomics revealed the regulatory role of manganese on small-molecule metabolism of biofilm formation in *escherichia coli*. J Anal Test..

[29] Guo R., Luo X., Liu J., Lu H. (2021). Mass spectrometry based targeted metabolomics precisely characterized new functional metabolites that regulate biofilm formation in *Escherichia coli*. Anal. Chim. Acta.

[30] Kuhnert W.L., Quivey R.G. (2003). Genetic and Biochemical Characterization of the F-ATPase Operon from *Streptococcus sanguis* 10904. J. Bacteriol..

[31] Xu W., Zhang X., Wang L., Zeng W., Sun Y., Zhou C., Zhou T., Shen M. (2022). Effect of chlorogenic acid on the quorum-sensing system of clinically isolated multidrug-resistant *Pseudomonas aeruginosa*. J. Appl. Microbiol..

[32] Heid C.A., Stevens J., Livak K.J., Williams P.M. (1996). Real Time Quantitative PCR. Genome Res..

[33] Majumdar S., Pal S. (2016). Quorum sensing: A quantum perspective. J. Cell Commun. Signal..

[34] Scoffone V.C., Trespidi G., Chiarelli L.R., Barbieri G., Buroni S. (2019). Quorum Sensing as Antivirulence Target in Cystic Fibrosis Pathogens. Int. J. Mol. Sci..

[35] Huang H., Shao X., Xie Y., Wang T., Zhang Y., Wang X., Deng X. (2019). An integrated genomic regulatory network of virulence-related transcriptional factors in *Pseudomonas aeruginosa*. Nat. Commun..

[36] Allesen-Holm M., Barken K.B., Yang L., Klausen M., Webb J.S., Kjelleberg S., Molin S., Givskov M., Tolker-Nielsen T. (2006). A characterization of DNA release in *Pseudomonas aeruginosa* cultures and biofilms. Mol. Microbiol..

[37] Yang L., Nilsson M., Gjermansen M., Givskov M., Tolker-Nielsen T. (2009). Pyoverdine and PQS mediated subpopulation interactions involved in *Pseudomonas aeruginosa* biofilm formation. Mol. Microbiol..

[38] Shakya S., Danshiitsoodol N., Noda M., Inoue Y., Sugiyama M. (2022). 3-Phenyllactic acid generated in medicinal plant extracts fermented with plant-derived lactic acid bacteria inhibits the biofilm synthesis of Aggregatibacter actinomycetemcomitans. Front. Microbiol..

[39] Zhang J., Wang D., Sun J., Sun Z., Liu F., Du L., Wang D. (2021). Synergistic Antibiofilm Effects of Ultrasound and Phenyllactic Acid against *Staphylococcus aureus* and *Salmonella enteritidis*. Foods.

[40] Giese A., Kerpen J., Weber F., Prediger J. (2021). A Preliminary Study of Microplastic Abrasion from the Screw Cap System of Reusable Plastic Bottles by Raman Microspectroscopy. ACS Es&T Water.

[41] Nakaya S., Yamamoto A., Kawanishi T., Toya N., Miyakawa H., Takeuchi K., Endo M. (2021). Detection of dynamic biofouling from adenosine triphosphate measurements in water concentrated from reverse osmosis desalination of seawater. Desalination..

[42] Keerthana Devi M., Karmegam N., Manikandan S., Subbaiya R., Song H., Kwon E.E., Sarkar B., Bolan N., Kim W., Rinklebe J. (2022). Removal of nanoplastics in water treatment processes: A review. Sci. Total Environ..

[43] Akther N., Sodiq A., Giwa A., Daer S., Arafat H.A., Hasan S.W. (2015). Recent advancements in forward osmosis desalination: A review. Chem. Eng. J..

[44] Su H., Liu Y., Pan C., Chen J., He L., Ying G. (2018). Persistence of antibiotic resistance genes and bacterial community changes in drinking water treatment system: From drinking water source to tap water. Sci. Total Environ..

[45] Sala-Comorera L., Blanch A.R., Vilaró C., Galofré B., García-Aljaro C. (2016). *Pseudomonas*-related populations associated with reverse osmosis in drinking water treatment. J. Environ. Manag..

[46] Almonacid Garrido M.C., Jiménez Navarro P., Peinador Asensio J., Villanueva Suárez M.J., Tenorio Sanz M.D. (2021). Tap Water Lead Levels in Madrid (Spain): Degree of Compliance and Health Risk Assessment. Expo. Health.

[47] Gambino I., Bagordo F., Grassi T., Panico A., De Donno A. (2022). Occurrence of Microplastics in Tap and Bottled Water: Current Knowledge. Int. J. Environ. Res. Public Health.

[48] Thapa K., Shrestha S.M., Rawal D.S., Pant B.R. (2019). Quality of drinking water in Kathmandu valley, Nepal. Sustain. Water Resour. Manag..

[49] Rather M.A., Gupta K., Mandal M. (2021). Microbial biofilm: Formation, architecture, antibiotic resistance, and control strategies. Braz. J. Microbiol..

[50] Kocot A.M., Olszewska M.A. (2017). Biofilm formation and microscopic analysis of biofilms formed by *Listeria monocytogenes* in a food processing context. LWT.

[51] Sudagidan M., Ozalp V.C., Öztürk O., Yurt M.N.Z., Yavuz O., Tasbasi B.B., Ucak S., Mavili Z.S., Coban A., Aydin A. (2021). Bacterial surface, biofilm and virulence properties of *Listeria monocytogenes* strains isolated from smoked salmon and fish food contact surfaces. Food Biosci..

[52] Lu L., Zhao Y., Yi G., Li M., Liao L., Yang C., Cho C., Zhang B., Zhu J., Zou K. (2021). Quinic acid: A potential antibiofilm agent against clinical resistant *Pseudomonas aeruginosa*. Chin. Med..

[53] Sycz Z., Tichaczek-Goska D., Wojnicz D. (2022). Anti-Planktonic and Anti-Biofilm Properties of Pentacyclic Triterpenes-Asiatic Acid and Ursolic Acid as Promising Antibacterial Future Pharmaceuticals. Biomolecules.

[54] Wang L., Li Y.X., Liu G.R. (2020). Scavenging Effect of *Honeysuckle* and *Dandelion* Extracts on the Biofilm of *Pseudomonas* Stains from Meat. Sci. Tech. Food Ind..

[55] Sun J.Y., Huang L.H., Sun Z.L., Wang D.B., Liu F., Du L.H., Wang D.Y. (2022). Combination of ultrasound and chlorogenic acid for inactivation of planktonic and biofilm cells of *Pseudomonas fluorescens*. Food Res. Int..

[56] Sun J., Wang D., Sun Z., Liu F., Du L., Wang D. (2021). The combination of ultrasound and chlorogenic acid to inactivate *Staphylococcus aureus* under planktonic, biofilm, and food systems. Ultrason. Sonochem..

[57] Angusamy A., Balasubramanian V., Arunmurugan B., Arunachalam K., Abraham S.V.P.I., Murugesan S., Krishnasamy B., Sundaram J., Arumugam V.R. (2023). Anti-infective potential of plant-derived quorum sensing inhibitors against multi-drug resistant human and aquatic bacterial pathogens. World J. Microbiol. Biotechnol..

[58] Wang L., Zhang Y., Liu Y., Xu M., Yao Z., Zhang X., Sun Y., Zhou T., Shen M. (2022). Effects of chlorogenic acid on antimicrobial, antivirulence, and anti-quorum sensing of carbapenem-resistant *Klebsiella pneumoniae*. Front. Microbiol..

[59] Okuyama K., Hamamoto T., Noguchi T., Midorikawa Y. (1996). Molecular cloning and expression of the pyrimidine nucleoside phosphorylase gene from *Bacillus stearothermophilus* TH 6-2. Biosci. Biotechnol. Biochem..

[60] Kao C.H., Hsu W.H. (2003). A gene cluster involved in pyrimidine reductive catabolism from *Brevibacillus agri* NCHU1002. Biochem. Biophys. Res. Commun..

[61] Nie M., Li K., Li Z. (2023). β-Alanine Metabolism Leads to Increased Extracellular pH during the Heterotrophic Ammonia Oxidation of *Pseudomonas putida* Y-9. Microorganisms.

[62] Hidese R., Mihara H., Kurihara T., Esaki N. (2011). *Escherichia coli* Dihydropyrimidine Dehydrogenase Is a Novel NAD-Dependent Heterotetramer Essential for the Production of 5,6-Dihydrouracil. J. Bacteriol..

[63] Kim G.J., Lee D.E., Kim H.S. (2000). Functional Expression and Characterization of the Two Cyclic Amidohydrolase Enzymes, Allantoinase and a Novel Phenylhydantoinase, from *Escherichia coli*. J. Bacteriol..

[64] Zhu D., Wei Y., Yin J., Liu D., Ang E.L., Zhao H., Zhang Y. (2020). A Pathway for Degradation of Uracil to Acetyl Coenzyme A in *Bacillus megaterium*. Appl. Environ. Microbiol..

