# Supplementary material for: Anti-Biofilm Activity of Chlorogenic Acid against Pseudomonas Using Quorum Sensing System
Source: Foods. 2023 Sep 28;12(19):3601. doi: 10.3390/foods12193601 (PMC10572673; doi:10.3390/foods12193601)
Supplement: Supplementary file 1 [file foods-12-03601-s001.zip › foods-2611121-supplementary.pdf]

Tables

Table S1 Results of VITEK Biochemical Identification

Biochemicals	401	411	412	433	439	509	513	621	623	647	656	671	694	695	696	707	710	717	861	862	874	886	20066
dTRE	+	−	−	−	+	−	−	−	−	−	−	−	+	−	−	−	−	−	+	−	−	−	+
CIT	−	+	+	+	+	+	+	+	+	+	+	+	+	+	+	+	+	+	+	+	+	+	+
MNT	−	−	−	+	+	+	+	+	+	+	−	−	+	−	+	−	+	+	+	+	+	+	+
5KG	−	−	−	−	−	−	−	−	−	−	−	−	−	−	−	−	−	−	−	−	−	−	−
lLATk	+	+	+	+	+	+	+	+	+	+	+	+	+	+	+	+	+	+	+	+	+	+	+
AGLU	−	−	−	−	−	−	−	−	−	−	−	−	−	−	−	−	−	−	−	−	−	−	−
SUCT	+	+	+	+	+	+	+	+	+	+	+	+	+	+	+	+	+	+	+	+	+	+	+
NAGA	−	−	−	−	−	−	−	−	−	−	−	−	−	−	−	−	−	−	−	−	−	−	−
AGAL	−	−	−	−	−	−	−	−	−	−	−	−	−	−	−	−	−	−	−	−	−	−	−
PHOS	+	−	−	−	−	−	−	−	−	−	−	−	−	−	−	−	−	−	−	−	−	−	−
GlyA	−	−	−	−	−	−	−	−	−	−	−	−	−	+	−	−	−	−	−	−	−	−	−
ODC	+	−	−	−	−	−	−	−	−	−	−	−	−	−	−	−	−	−	−	−	−	−	−
LDC	−	−	−	−	−	−	−	−	−	−	−	−	−	−	−	−	−	−	−	−	−	−	−
lHISa	−	+	+	+	−	−	−	+	−	−	+	−	−	−	−	+	+	−	−	−	−	−	+
CMT	+	+	+	+	+	+	+	+	+	+	+	+	+	+	+	+	+	+	+	+	+	+	+
BGUR	−	−	−	−	−	−	−	−	−	−	−	−	−	−	−	−	−	−	−	−	−	−	−
O129R	+	+	+	+	+	+	+	+	+	+	+	+	+	+	+	+	+	+	+	+	+	+	+
GGAA	−	−	−	−	−	−	−	−	+	−	−	−	−	−	−	−	−	−	−	(−)	+	−	−
lMLTa	−	+	+	+	+	+	+	+	+	+	+	+	−	−	+	+	+	+	+	+	+	+	+
ELLM	+	−	−	−	−	−	−	−	−	−	−	−	−	−	−	−	−	−	−	−	−	−	−
lLATa	−	+	−	−	+	+	+	+	−	+	+	−	−	−	−	−	+	+	−	−	+	+	+
Results	A	A	A	A	B	B	B	A	B	B	A	A	B	C	A	A	A	B	B	B	B	B	B
Confidence%	99	99	9	99	97	95	99	99	95	99	99	99	93	98	99	99	99	99	97	99	95	99	93

For the letters A-C means, A: *Pseudomonas putida*; B: *Pseudomonas aeruginosa*; C: *Pseudomonas fluorescens.*
The full name of the biochemicals abbreviation can be seen from the VITEK GN operation manual.

Table S2 Peak areas of metabolites by GC-MS analysis

Class	RT(min)	Description	Non-CGA	8.47mM CGA	16.93mM CGA	25.4mM CGA
Sulfur compound	6.1961	Disulfide, dimethyl	2534.50±2.12	2551.00±22.63	2537.50±0.71	2533.50±71.00
	13.9153	Dimethyl trisulfide	11081.00±189.50	28.00±9.90	31.00±5.66	65.50±2.12
	28.0576	S-Ethyl ethanethioate	4855.50±178.90	14.00±1.41	14.00±2.83	22.00±1.41
Amide	7.2166	L-Asparagine	23015.00±155.56	691.50±55.86	1006.00±26.87	4888.50±89.80
	17.9102	Auramine	116332.50±30.41	116332.50±30.41	116483.50±	116594.50±
	28.7491	Methylamine, N,N-dimethyl-	253.50±0.71	253.50±0.71	254.00±39.30	253.50±43.13
	31.4433	Isobutylamine	762.50±17.68	15326.50±419.31	127871.00±7629.68	8763.50±185.97
	31.1144	Methanethioamide,N,N-dimethyl-	2218.50±50.20	14.00±1.41	18.00±1.41	21.00±1.41
	32.933	1-Propanamine, N-ethyl-	2187.00±36.77	71.00±5.66	557.00±15.56	1964.50±68.59
	33.3682	1-Butanamine, 3-methyl-	2185.00±39.60	1938.50±24.75	1974.00±1.41	1985.50±716.00
	33.494	Ethanamine, N-methyl-	273.50±30.41	1996.50±34.65	780.00±49.50	300.50±16.26
	29.7988	N-Methyl-4-pyridinamine	5315.00±300.00	5315.00±320.00	5315.00±53.15	5315.00±563.10
	35.3417	Ethanamine, N-methyl-	13.50±3.54	9171.50±65.76	1992.50±28.99	273.50±30.41
	35.6754	1-Butanamine, N-methyl-	14.00±1.41	2029.50±149.20	14.00±1.41	18.00±1.41
	30.4905	1-Methyl-4-amino-4,5(1H)-dihydro-1,2,4-triazole-5-one	7111.50±75.66	12.00±1.41	14.50±3.54	23.50±3.54
	30.6017	Aminoacethydrazide	2203.00±87.68	16.00±1.41	12.50±0.71	12.00±1.41
	36.1638	Indole	8576.00±650.00	8576.00±680.00	8576.00±867.52	8576.00±578.23
	32.3042	Ethanone, 1-(2-aminophenyl)-	15525.50±710.00	15527.00±163.75	15527.00±156.43	15527.00±168.72
	33.6488	.beta.-Alanine	2186.50±37.48	93.00±2.83	2072.50±459.60	217219.00±25139.97
Alkene	7.4583	1-Undecene	27336.00±449.75	27070.50±1.42	27042.50±3182.00	27016.50±4.95
	15.7726	2-Pentene, 4,4-dimethyl-, (E)-	3402.50±49.50	1336.00±39.60	1382.00±117.38	72928.50±85873.17
	22.1763	2,6-Octadien-1-ol, 2,7-dimethyl-	106648.00±6655.29	27212.00±625.08	10300.00±70.71	5335.50±126.57

	22.9309	11-Dodecen-2-one	144888.50±2310.12	143412.50±2840.45	88986.00±743.88	15326.50±419.31
Esters	9.0448	Butanethioic acid, S-methyl ester	8813.00±67.88	7992.50±48.79	6025.50±36.06	597.50±3.54
	9.4705	S-Methyl 3-methylbutanethioate	14372.00±19.80	14369.50±21.92	14371.50±19.09	14373.50±21.92
	9.688	Thiovaleric acid S-propyl ester	14662.00±429.92	23508.00±2763.37	43659.00±1211.98	31980.50±939.74
	11.5309	Formic acid, 2-methylpentyl ester	1499.50±19.09	7783.50±327.39	99495.00±10807.4	13697.00±275.77
	19.8886	Octanethioic acid, S-methyl ester	42104.00±582.66	22.00±1.41	18.00±1.41	26.00±1.41
Alcohol	10.0219	1-Pentanol	11962.50±983.59	597.50±20.51	613.50±13.44	618.50±33.23
	16.2079	1-Heptanol	8617.00±421.44	20971.50±119.50	25406.50±383.96	30590.50±614.48
	17.0398	1-Hexanol, 2-ethyl-	13668.50±0.71	13666.00±7.07	13672.50±16.26	13672.00±4.24
	17.8088	2-Nonanol	21100.50±2.12	21089.50±0.71	21083.50±4.95	210990.50±2.12
	22.5052	2-Dodecanol	98615.00±3727.87	50667.00±503.46	85422.50±1171.68	4462.50±75.66
	20.6526	Cyclohexanol	13264.50±67.18	28390.00±5.66	28386.50±0.71	28353.00±16.97
	21.949	L-.alpha.-Terpineol	27084.00±142.84	16099.00±601.04	26880.00±147.08	26719.50±48.79
	24.1109	Ethanol, 2-(2-butoxyethoxy)-	32655.00±1.41	32656.50±2.12	32658.50±0.71	32657.00±2.83
	25.7361	Benzyl alcohol	132350.00±5461.69	5535.50±231.22	26005.00±763.68	86337.00±869.74
	26.4712	Phenylethyl Alcohol	9921.00±0.00	9921.00±0.00	9921.50±0.71	9921.00±0.00
	29.2523	(3,3-Dimethyloxiranyl)methanol	4426.50±140.71	22.00±1.41	33.00±2.83	14.00±1.41
	34.1421	Glycerol	2420.00±0.00	2420.50±0.71	2420.50±0.71	2420.50±0.71
Pyrazine	11.3035	Pyrazine, methyl-	2593.50±30.41	13.50±2.12	14.00±2.83	15.50±0.71
	12.8029	Pyrazine, 2,5-dimethyl-	5316.00±7.07	5317.00±1.41	5318.00±1.41	5318.50±3.54
	14.5441	Pyrazine, 2-ethyl-6-methyl-	9839.50±7.78	9830.00±2.83	9844.00±2.83	9831.00±4.24
	14.9407	Pyrazine, trimethyl-	9812.00±1.41	9814.00±1.41	9813.50±2.12	9812.00±1.41
	16.353	Pyrazine, 2-ethyl-3,5-dimethyl-	15705.00±394.57	16292.50±84.15	12956.50±409.41	15550.00±193.75
	21.1074	2-(3-Methylbutyl)-3,5-dimethylpyrazine	44077.50±187.38	8385.50±94.05	5931.00±97.58	2500.00±206.48
Alkyne	13.5623	3-Heptyne	35684.50±750.24	2835.00±0.00	2869.00±21.21	2870.00±19.8
Ketone	14.3021	2-Nonanone	19938.00±0.00	19938.00±0.00	19938.00±0.00	19938.00±0.00

	19.608	2-Undecanone	38224.00±0.00	38224.00±0.00	38224.00±0.00	38227.00±4.24
	24.4204	2-Tridecanone	71829.50±738.90	59952.50±243.90	60415.00±862.67	60105.50±348.60
	27.9851	5H-1,2,4-Triazolo[4,3-b][1,2,4]triazepine, 6,7,8,9-tetrahydro-6-methyl-	27054.50±1112.20	28.50±3.50	43.50±21.92	22.00±14.14
Aldehyde	15.3517	2-Octenal, (E)-	11731.00±565.60	558.00±29.70	3377.00±39.60	11682.50±497.10
	17.6396	Benzaldehyde	5067.00±0.00	5067.00±0.00	5067.00±0.00	5067.00±0.00
Organic acid	15.9902	Acetic acid	264.00±1.41	263.50±0.71	263.50±2.12	263.50±0.71
	18.1329	Propanoic acid	813.00±2.83	813.00±1.41	814.00±2.83	815.00±
	25.0928	Hexanoic acid	8071.00±0.00	8071.00±0.00	8071.00±0.00	8071.00±0.00
	25.8279	Pentanoic acid, 2,2,4-trimethyl-3-carboxyisopropyl, isobutyl ester	132857.00±0.00	132857.00±0.00	132856.50±0.71	132857.00±0.00
	25.6249	Propanoic acid, 2-methyl-, hexyl ester	12.00±1.41	39731.50±184.55	26194.50±499.92	21275.50±535.28
	27.2788	Hexanoic acid, 2-ethyl-	2093.50±21.92	141443.00±5774.23	53622.00±1414.21	21460.50±846.41
	29.4263	Octanoic acid	17027.00±185.26	28637.50±731.86	20952.50±163.34	154686.50±179079.74
Alkane	17.2478	Octane	24344.00±335.17	3982.00±42.43	5498.50±65.76	7715.50±136.40
	31.274	Cyclopropane, (methoxymethyl)-	1869.00±73.54	14.50±2.12	13.00±2.83	18.00±1.41
Heterocycle	18.776	Aziridine, 2,2-dimethyl-	13660.50±48.70	3399.00±849.00	627.50±10.60	8882.00±53.74
	23.4145	Oxime-, methoxy-phenyl-_	24837.00±0.00	24837.00±0.00	24837.00±0.00	24837.00±0.00
	29.1459	1,6-Octadiene, 8-methoxy-	19255.50±772.87	12.00±1.41	14.00±1.41	18.00±1.41
	35.9558	6-Propyltetrahydro-2H-thiopyran-2-one	241992.00±5176.02	28533.00±732.56	30570.00±644.88	43245.50±447.60
Aromatic compounds	28.2947	Phenol	2593.00±0.00	2593.00±0.00	2593.00±0.00	2593.00±0.00
	30.234	Butylated Hydroxytoluene	65711.50±579.12	65628.50±463.15	77504.00±91.92	77757.00±285.67
Sulfur compound	6.1961	Disulfide, dimethyl	2534.50±2.12	2551.00±22.63	2537.50±0.71	2533.50±0.71
